# Coffee Consumption and Risk of Colorectal Cancer: A Systematic Review and Meta-Analysis of Prospective Studies

**DOI:** 10.3390/nu11030694

**Published:** 2019-03-24

**Authors:** Marina Sartini, Nicola Luigi Bragazzi, Anna Maria Spagnolo, Elisa Schinca, Gianluca Ottria, Chiara Dupont, Maria Luisa Cristina

**Affiliations:** 1 Department of Health Sciences (DISSAL), University of Genoa, 16132 Genoa, Italy; am.spagnolo@unige.it (A.M.S.); elisa.schinca@unige.it (E.S.); gianluca.ottria@unige.it (G.O.); lioa@unige.it (C.D.); cristinaml@unige.it (M.L.C.); 2 Postgraduate School of Public Health, Department of Health Sciences (DISSAL), University of Genoa, 16132 Genoa, Italy; robertobragazzi@gmail.com

**Keywords:** coffee/caffeine, systematic review and meta-analysis, prospective studies, epidemiology, cancer prevention, colorectal cancer

## Abstract

Coffee is a blend of compounds related to gastrointestinal physiology. Given its popularity and the epidemiology of colorectal cancer, the impact of this beverage on public health could be considerable. Our aim was to provide an updated synthesis of the relationship between coffee consumption and the risk of colorectal cancer. We conducted a systematic review and meta-analysis of 26 prospective studies. Regarding colorectal cancer, no significant relationship was detected. Stratifying for ethnicity, a protective effect emerged in US subjects. Concerning colon cancer, coffee proved to exert a protective effect in men and women combined and in men alone. Stratifying for ethnicity, a significant protective effect was noted in European men only and in Asian women only. Concerning rectal cancer, no association was found. Decaffeinated coffee exhibited a protective effect against colorectal cancer in men and women combined. Studies were appraised for their quality by means of the Newcastle-Ottawa Quality Assessment Scale for Cohort studies. Only one study proved to be of low quality. Ethnicity could explain the heterogeneity of the studies. However, little is known about the relationship between the genetic make-up and the risk of colorectal cancer associated with coffee. Further research is warranted.

## 1. Introduction

Coffee is a complex blend of bioactive compounds. These are related to gastrointestinal physiology in various ways and may exert contrasting effects. On the one hand, coffee contains anti-oxidants and anti-mutagens, which could, as such, reduce exposure of the epithelial cells of the bowel to carcinogenic chemicals and compounds, and prevent and counteract the effect of potential promoters of intestinal carcinogenesis. Indeed, coffee seems to enhance bowel motility and functioning, reducing fecal transit times and increasing stool output. Furthermore, coffee contains lipidic compounds, such as cafestol and kahweol, which, by finely tuning cholesterol metabolism, reduce the synthesis and release of bile acids [1]. They act also as reactive oxygen species (ROS) scavengers, activating DNA repair enzymes [2] and phase-II enzymes involved in carcinogen detoxification. Furthermore, coffee modulates the microbiome of the gut. Caffeine has anti-apoptotic effects, inducing programmed cell death [3]. Caffeic acid is involved in several pathways related to inflammation, apoptosis and the cell cycle. Finally, chlorogenic acid seems to have antioxidant properties. On the other hand, coffee contains mutagens, such as glyoxal, methylglyoxal, ethylglyoxal, propylglyoxal, diacetyl, acetol and other dicarbonyls [4,5,6,7] or tannins [8], among others, which could counteract the protective effects of coffee.

Moreover, the quantity of these bioactive compounds varies according to the type of coffee. For example, unfiltered coffees, such as French press coffee or Turkish/Greek coffee, contain significant amounts of these compounds, unlike filtered blends, such as dip-brewed coffee. Moreover, filtered coffee also contains fewer phenols and polyphenols [2,9].

Being cheap and easy to prepare, coffee is a widespread beverage, its worldwide consumption of two billion cups per day being second only to that of water [2]. Given the popularity of coffee, its impact on public health could be considerable. Statistics on cancer reveal that colorectal cancer is the third most common cancer among men, after lung and prostate cancer, and the second most common cancer among women, after breast cancer; in terms of cancer-related death, it ranks fourth after lung, liver and stomach cancer [10]. We therefore aimed to provide an updated quantitative synthesis of the relationship between coffee consumption and the risk of colorectal cancer.

## 2. Materials and Methods 

The Preferred Reporting Items for Systematic Reviews and Meta-analyses (PRISMA) guidelines [11] were used as a guide to ensure that the current standard for meta-analysis methodology were met (see also Supplementary Materials). We searched PubMed/MEDLINE and Scopus archives and databases for a combination of keywords such as “coffee” and “colorectal cancer”, using Medical Subject Headings (MeSH) terms as vocabulary, according to the National Center for Biotechnology Information (NCBI) nomenclature and guidelines and, where appropriate, a wild-card option. 

Inclusion criteria were: (1) articles with relevant quantitative details and information on the relationship between coffee consumption and the risk of developing colorectal cancer; (2) prospective studies. Exclusion criteria were: (1) items not directly pertinent to the query string; (2) articles not containing sufficient information on the relationship between coffee consumption and the risk of colorectal cancer; (3) articles not meeting the PICOS criteria (P: patients with colorectal cancer; I: consuming coffee; C: coffee consumption versus non-consumption, and/or comparison between different kinds of coffee: caffeinated or decaffeinated, etc.; O: risk ratio, RR, of colorectal cancer associated to coffee consumption; S: prospective study); all such articles were consequently discarded. No time filter or language filter was applied. For further details of the search strategy, see Table 1.

Two of the authors independently screened the literature. Any case of disagreement was solved by discussion until consensus was reached. After the full text review, the papers included were retained for data extraction.

Data for the meta-analysis were extracted from the studies included by means of a standardized documentation form. The parameters extracted were: the surname of the first author, the year and country of publication, sample size, percentages of females and males, incidence of colorectal cancer (broken down by clinical site: colon and rectum), and the amount and type of coffee consumed. 

RR of developing colorectal cancer on the basis of coffee consumed (i.e., RR of subjects with colorectal cancer who consumed the greatest amount of coffee versus subjects who did not consume coffee) were calculated as effect size estimates, with their 95% confidence intervals (CIs). Additional analyses were performed after stratification by type of coffee, study region, publication period and gender. 

Study quality was appraised by two researchers, working independently, with respect to the appropriateness of the research questions tested and of the methods employed. For this purpose, the Newcastle-Ottawa Quality Assessment Scale for Cohort studies (NOS) was used; scores of ≥7 indicated high-quality and <7 indicated low-quality studies. Any disagreement was solved by consensus. 

Statistical heterogeneity was also assessed in our meta-analysis by means of I^2^ statistics and chi-square test, heterogeneity being deemed significant if the *p* value (χ^2^) was <0.1. In detail, it was determined that the values of 25%, 50% and 75% in the I^2^ test corresponded to low, moderate and high levels of heterogeneity, respectively. In the event of significant (moderate or high) heterogeneity among the studies, a random-effects model was used for the meta-analysis. The RR of the meta-analyses were deemed significant when the confidence intervals did not contain the value “1”; indeed, if the confidence interval contains the value “1”, we cannot exclude the absence of an association between exposure and disease. A narrower CI than that of the individual studies indicates less imprecision. 

Meta-analyses were carried out by means of the STATA SE14^R^ (StataCorp LP, College Station, TX, USA) software. To identify sources of variation, further stratification was performed with respect to study quality. In addition, in the sensitivity analyses, the stability of the pooled estimate with respect to each study was investigated by excluding individual studies from the analysis. Possible publication bias was visually inspected by means of a funnel plot. If asymmetry was detected by visual assessment, exploratory analyses using trim and/or fill analysis were performed in order to investigate and adjust the effect-size estimate. In addition, the probability of publication bias was tested by means of Egger’s linear regression, a value of *p* < 0.05 being indicative of publication bias.

## 3. Results

**Figure 1:**
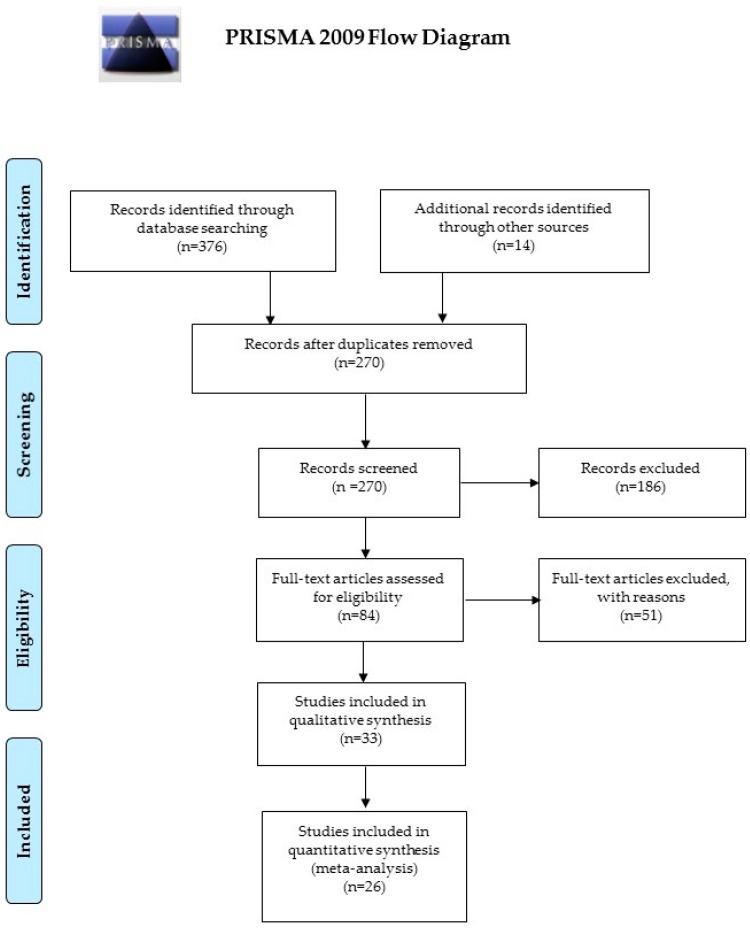
Flowchart of study selection, inclusion and synthesis.

Concerning the systematic review, our initial query resulted in 390 hits (specifically, 376 articles from PubMed/MEDLINE and Scopus, and 14 from other sources); after removal of duplicate items, the resulting list comprised of 270 non-redundant articles. Only 33 studies were retained in the qualitative synthesis, and 26 were finally considered in our systematic review and meta-analysis (186 articles were discarded as not being directly pertinent to the topic under investigation and 51 as not meeting the inclusion criteria). For further details, see Figure 1.

The full list of studies included [12,13,14,15,16,17,18,19,20,21,22,23,24,25,26,27,28,29,30,31,32,33,34,35,36,37] and their main characteristics are shown in Table 2. 

The studies examined included 3,308,028 subjects. Eleven studies were performed in European countries, seven in Asian countries and seven in the USA. Nineteen studies were on colorectal cancer, 19 on colon cancer and 18 on rectal cancer.

With regard to colorectal cancer, from the pooled RR, no significant relationship between coffee consumption and the risk of developing cancer was detected (Table 3). 

Stratifying for ethnicity, a significant protective effect emerged among US subjects (men and women), with a RR of 0.83 (95% CI 0.72–0.95). While no statistical significance emerged for caffeinated coffee, decaffeinated coffee exhibited a protective effect on men and women combined (RR 0.88 (95% CI 0.78–0.97)).

**Figure 2:**
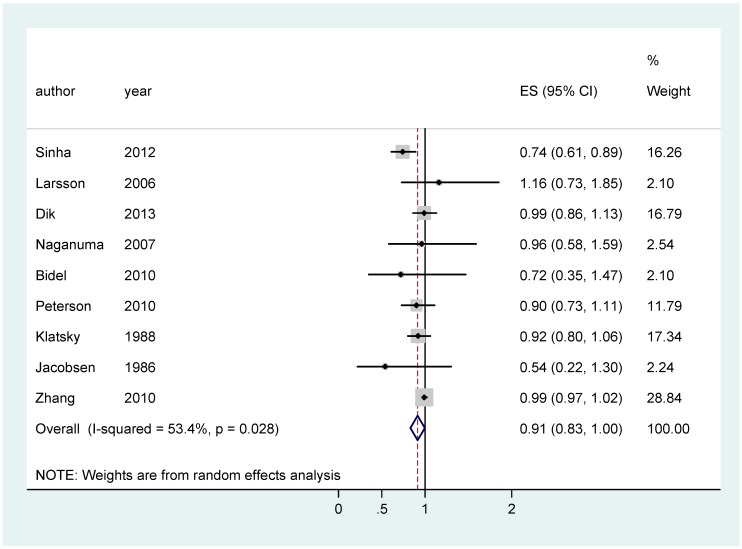
Forest plot of colon cancer in men and women combined.

**Figure 3:**
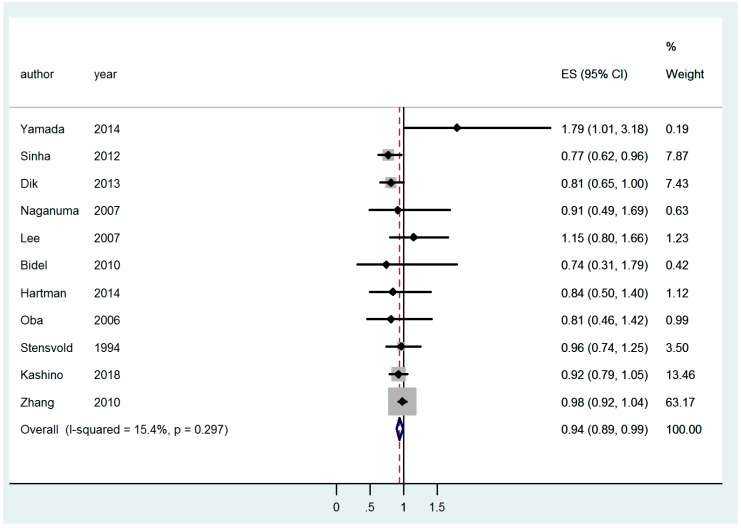
Forest plot of colon cancer in men.

Concerning colon cancer (Table 3), coffee consumption proved to exert a protective effect on men and women combined (RR 0.91 (95% CI 0.83–0.998)) (Figure 2), and on men only (RR 0.94 (95% CI 0.89–0.99)) (Figure 3). 

**Figure 4:**
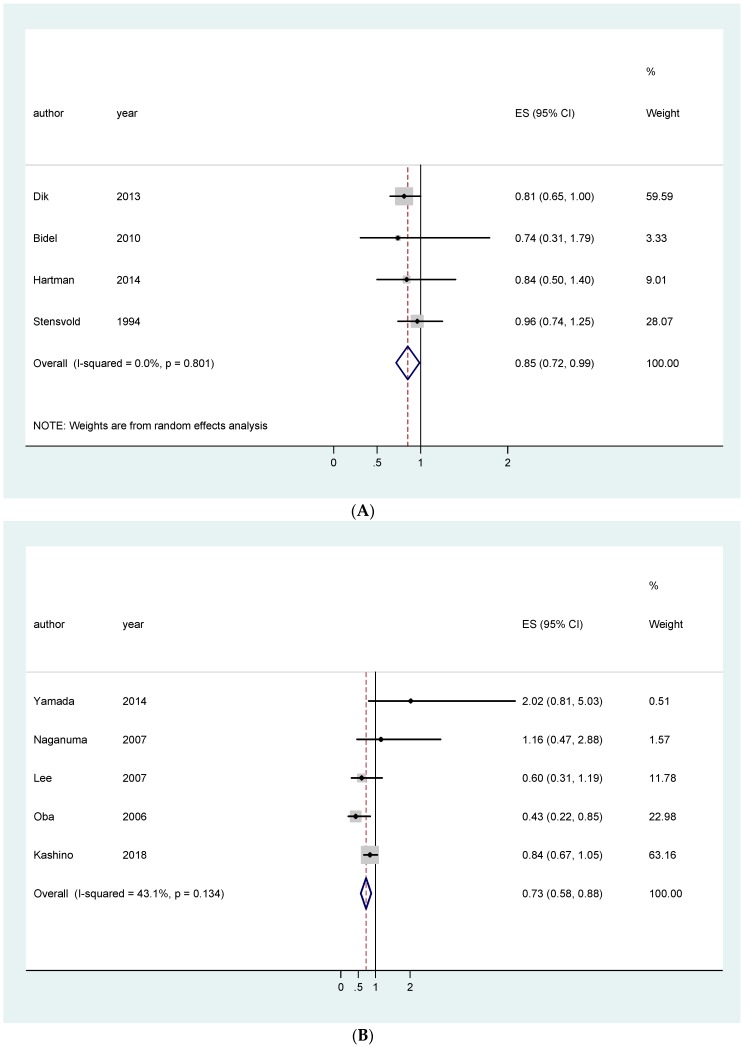
Forest plot of colon cancer in European men (**A**) and in Asian women (**B**).

Stratifying for ethnicity, a statistically significant protective effect was noted in European men only (RR 0.85 (95% CI 0.72–0.99)), and in Asian women only (RR 0.73 (95% CI 0.58–0.88)) (Figure 4A,B).

Focusing on distal colon cancer (Table 3), coffee consumption proved protective in European men only (RR 0.77 (95% CI 0.57–0.98)). With regard to proximal colon cancer (Table 3), no significant association was found. 

Concerning rectal cancer (Table 3), no significant association could be found.

No significant publication bias was detected. Finally, concerning the assessment of the risk of bias, no significant biases emerged. Only one study was deemed to be of low quality (Wu: NOS score = 5). Retaining or removing this study from the meta-analyses did not change the results.

## 4. Discussion

Performing the meta-analysis of prospective studies, we found a significantly high degree of heterogeneity. 

With specific regard to rectal cancer, no evidence of an association between coffee consumption and the development of the disease could be found. For colorectal cancer, we found evidence of a protective effect only in US men and women together. Stratification by type of coffee—caffeinated or decaffeinated—did not reveal any differences linked to the presence of caffeine, since the types of coffee appeared to be protective in some studies and non-significant in others. Moreover, the quantity of caffeine in coffee also depends on the type of mixture used [38], a parameter that was not investigated in the studies considered. Instead, in the present systematic review and meta-analysis decaffeinated coffee consumption showed a protective role against colorectal cancer, despite the low number of investigations.

Regarding colon cancer, evidence of a protective effect of coffee consumption was found, both considering men and women together and considering men alone. 

Stratifying for ethnicity, we found that the pooled RR was significant for European men and for Asian women. We can speculate that this might be due to the particular type of coffee consumed; for example, in Asia, coffee is rarely consumed boiled or decaffeinated [22]. Another possible explanation may lie in the biological make-up of the subjects; this might also explain why coffee drinking proved protective in a particular gender but not in the other. In this regard, Platt et al. found that cultural, dietary and lifestyle factors influenced the impact of coffee intake in terms of the risk of developing metabolic syndrome [39]. Similarly, Kumar et al. found a differential effect of caffeine intake on the onset of Parkinson’s disease, the effect being mediated by genetic variants [40]. Other biological events or diseases have been found to be characterized by a complex gene–caffeine interaction, such as variability in the cardiovascular response to caffeine [41], coffee consumption and the risk of developing neurodegenerative disorders [42], epithelial ovarian cancer [43], breast cancer [44], or myocardial infarction [45], among others. These genes include cytochrome P450 1A2 (CYP1A2), adenosine A2a receptor (ADORA2A), and leucine-rich repeat kinase 2 or dardarin (LRRK2). 

As potential mechanisms that may explain the inverse association between coffee consumption and the development of colon cancer, a review by Higdon [46] hypothesizes the presence of diterpenes as a factor of protection. Indeed, in vitro studies have reported that this molecule is able to reduce the formation of DNA adducts by several genotoxic carcinogens, including 2-amino-1-methyl-6-phenylimidazo[4,5-b]pyridine (PhIP), a heterocyclic amine implicated in colon carcinogenesis. Moreover, it would seem that diterpenes can promote the elimination of carcinogens and improve antioxidant status. 

A major limitation of the present meta-analysis lies in the fact that most studies did not specify whether the coffee blend was caffeinated or decaffeinated, filtered or unfiltered, thus hindering the possibility of precisely stratifying the pooled RR according to the type of coffee consumed.

A major difficulty in interpreting epidemiological data is that studies often do not clearly indicate the quantity of coffee consumed (which is usually expressed as cups/day and not in mL); estimates are therefore “rough”. 

Moreover, the considerable variability in the composition of the beverage makes it difficult to accurately determine the potential quantity of bioactive substances involved in the process.

## 5. Conclusions

The development of colorectal carcinoma is a complex, multi-step process characterized by a series of both genetic and epigenetic changes, which involve different cellular cascades and pathways, including DNA repair, proliferation, apoptosis, intra- and extracellular signaling, and adhesion, among others [47]. Ethnicity seems to be an important variable in the relationship between coffee consumption and the risk of developing colorectal cancer. However, little is known about the relationship between genetic make-up and the risk of colorectal cancer associated with coffee consumption. Furthermore, given the above-mentioned limitations, further research in the field is warranted.

In conclusion, the available studies are not sufficient to define a protective role of coffee against colorectal cancer.

## Figures and Tables

**Table 1:** Search strategy adopted in the present systematic review and meta-analysis.

Search Strategy	Details
Search string	(coffee OR caffeine) AND (tumor OR cancer OR neoplasm) AND (colon OR rectal OR colorectal)
Databases	PubMed/MEDLINE, Scopus
Inclusion criteria	P (patients/population): general population/patients suffering from colorectal cancer I (intervention/exposure): subjects consuming coffee C (comparisons/comparators): coffee consumers versus non-consumers; different kinds of coffee (caffeinated/decaffeinated) O (outcome): incidence of colorectal cancer S (study design): prospective study
Exclusion criteria	Experimental studies investigating in vitro or animal models Study design: editorial, commentaries, expert opinions, letters to editor, review articles, original non-prospective studies, articles with insufficient details
Time filter	None (from inception)
Language filter	None (any language)

**Table 2:** Characteristics of studies included.

First Authors (year)	Country	Study Subject	Coffee Consumption (“high” vs. “low”)	No. Cases
**Jacobsen (1986) [12]**	Sweden	All (16,555) F (2891); M (13,664)	≥7 cups/d vs. ≤2 cups/d	97 CC—63 RC
**Wu (1987) [13]**	USA	All (11,632) F (7456); M (4163)	≥4 cups/d vs. ≤1 cup/d	NA (CRC)
**Klatsky (1988) [14]**	USA	All (10,572)	Continuous variable (cups/d)	203 CC—66 RC
**Stensvold (1994) [15]**	Sweden	F (21,238); M (21,735)	≥7 cups/d vs. ≤2 cups/d	F: 52 CC—38 RC; M: 78 CC—41 RC
**Terry (2001) [16]**	Sweden	F (61,463)	≥4 cups/d vs. <1 cup/d	291 CC—159 RC—460 CRC
**Khan (2004) [17]**	Japan	F (1634); M (1524)	Continuous variable (cups/d)	F: 14 CRC; M: 15 CRC
**Michels (2005) [18]**	USA	All (133,893) F (87,794); M (46,099)	>5 cups/d vs. none (caffeinated, decaffeinated)	Caffeinated: 1170 CC—260 RC—1431 CRC Decaffeinated: 913 CC—224 RC—1138 CRC
**Larsson (2006) [19]**	Sweden	All (106,739)	≥4 cups/d vs. <1 cup/d	843 CC—440 RC—1279 CRC
**Mucci (2006) [20]**	Sweden	F (61,467)	≥4 cups/d vs. ≤1 cup/d	504 CC—237 RC—741 CRC
**Oba (2006) [21]**	Japan	F (16,327); M (13,894)	≥1 cups/d vs. none	F: 102 CC; M: 111 CC
**Lee (2007) [22]**	Japan	F (50,139); M (46,023)	≥3 cups/d vs. none	F: 286 CC—151 RC—437 CRC M: 174 CC—102 RC—276 CRC
**Naganuma (2007) [23]**	Japan	All (47,605) F (24,769); M (22,836)	≥3 cups/d vs. none	ALL: 281 CC—180 RC—457 CRC F: 106 CC—68 RC—173 CRC M: 175 CC—112 RC—284 CRC
**Bidel (2010) [24]**	Finland	All (60,041) F (30,882); M (29,159)	≥10 cups/d vs. none	ALL: 333 CC—252 RC—538 CRC F: 167 CC—123 RC—271 CRC M: 166 CC—129 RC—267 CRC
**Nilsson (2010) [25]**	Sweden	All (64,603)	≥4 cups/d vs. <1 cup/d	321 CRC
**Simons (2010) [26]**	Netherlands	F (62,573) M (58,279)	>6 cups/d vs. ≤2 cups/d	F: 173 RC—939 CRC M: 322 RC—1260 CRC
**Zhang (2010) [27]**	Multi-center (conducted in USA and in Europe)	All (731,441)	High quintile vs. low quintile	5,604 CC
**Sinha (2012) [28]**	USA	All (489,706)	≥6 cups/d vs. none (all, caffeinated, decaffeinated)	5,072 CC—2863 Prox CC—1993 Distal CC—1874 RC—6946 CRC
**Dominianni (2013) [29]**	USA	All (57,398)	≥4 cups/d vs. none	681 CRC
**Perrigue (2013) [30]**	USA	All (67,912)	≥7 cups/d vs. <7 cups/d	409 CRC
**Dik (2014) [31]**	EPIC study (Europe)	All (521,448) F (365,014); M (156,434)	High quintile vs. low quintile (all, caffeinated, decaffeinated)	2691 CC—1242 Prox CC—1202 Distal CC—1543 RC—4234 CRC
**Hartmann (2014) [32]**	Finland	M (27,111)	>6 cups/d vs. ≤4 cups/d	106 CC—79 RC
**Peterson (2014) [33]**	Singapore	All (61,321)	≥2 cups/d vs. <1 cup/d	591 CC—370 RC
**Yamada (2014) [34]**	Japan	F (34,614) M (23,607)	≥4 cups/d vs. <1 cup/d	F: 332 CC—112 RC—444 CRC M: 355 CC—202 RC—557 CRC
**Groessl (2016) [35]**	USA	F (83,972)	≥4 cups/d vs. none	1,083 CC—160 RC—12,852 CRC
**Lukic (2016) [36]**	Norway	F (79,461)	>7 cups/d vs. ≤1 cup/d	1266 CRC
**Kashino (2018) [37]**	Japan	F (170,388) M (149,934)	≥3 cups/d vs. <1 cup/d	F: 1963 CC—770 RC—2689 CRC M: 2619 CC—1402 RC—4022 CRC

Abbreviations: CC (colon cancer); CRC (colorectal cancer); d (day); F (female); M (male); NA (not available); prox (proximal); RC (rectal cancer); vs. (versus).

**Table 3:** Risk ratio (RR) and 95% CI for all meta-analyses carried out. Values in bold are statistically significant.

*Tumor and Geographic Provenience of the Studies*		Men and Women	Men	Women
		RR [95%CI]; (N. Studies)	RR [95%CI]; (N. Studies)	RR [95%CI]; (N. Studies)
***CRC***	All	0.96 [0.88–1.03]: (8)	0.96 [0.88–1.04]; (9)	1.06 [0.97–1.14]; (13)
	EU studies only	1.07 [0.96–1.17]; (4)	0.93 [0.80–1.06]; (3)	1.10 [0.98–1.22]; (6)
	Asian studies only	NA	0.97 [0.87–1.08]; (5)	0.94 [0.78–1.09]; (5)
	USA studies only	**0.83 [0.72–0.95]; (3)**	NA	1.14 [0.92–1.36]; (2)
	Caffeinated coffee	0.96 [0.77–1.17]; (3)	NA	NA
	Decaffeinated coffee	**0.88 [0.78–0.97]; (3)**	NA	NA
***CC***	All	**0.91 [0.83–0.998]; (9)**	**0.94 [0.89–0.99]; (11)**	0.92 [0.80–1.03]; (13)
	EU studies only	0.96 [0.84–1.09]; (4)	**0.85 [0.72–0.99]; (4)**	1.05 [0.93–1.18]; (5)
	Asian studies only	0.91 [0.73–1.09]; (2)	0.94 [0.82–1.06]; (5)	**0.73 [0.58–0.88]; (5)**
	USA studies only	0.83 [0.66–1.01]; (2)	NA	0.90 [0.38–1.42]; (2)
	Caffeinated coffee	0.92 [0.68–1.15]; (3)	NA	NA
	Decaffeinated coffee	0.93 [0.81–1.05]; (3)	NA	NA
***Distal CC***	All	0.98 [0.95–1.02]; (5)	0.94 [0.87–1.01]; (5)	1.00 [0.96–1.04]; (6)
	EU studies only	NA	**0.77 [0.57–0.98]; (2)**	1.09 [0.82–1.36]; (3)
	Asian studies only	NA	0.83 [0.60–1.05]; (2)	0.91 [0.56–1.25]; (2)
	USA studies only	0.88 [0.65–1.12]; (2)	NA	NA
	Caffeinated coffee	0.99 [0.79–1.91]; (2)	NA	NA
	Decaffeinated coffee	1.05 [0.79–1.32]; (2)	NA	NA
***Proximal CC***	All	0.93 [0.73–1.15]; (5)	0.98 [0.92–1.04]; (5)	0.99 [0.96–1.03]; (6)
	EU studies only	NA	0.90 [0.66–1.14]; (2)	1.17 [0.90–1.44]; (3)
	Asian studies only	NA	1.08 [0.83–1.32]; (2)	0.85 [0.57–1.13]; (2)
	USA studies only	0.92 [0.23–1.60]; (2)	NA	NA
	Caffeinated coffee	0.85 [0.32–1.36]; (2)	NA	NA
	Decaffeinated coffee	0.86 [0.66–1.06]; (2)	NA	NA
***RC***	All	1.00 [0.89–1.11]; (9)	1.01 [0.87–1.14]; (9)	1.08 [0.94–1.23]; (11)
	EU studies only	1.17 [0.97–1.37]; (4)	0.96 [0.78–1.14]; (5)	1.04 [0.87–1.21]; (6)
	Asian studies only	1.04 [0.79–1.29]; (2)	1.07 [0.85–1.29]; (4)	1.28 [0.96–1.60]; (4)
	USA studies only	0.88 [0.72–1.04]; (3)	NA	NA
	Caffeinated coffee	1.18 [0.98–1.38]; (3)	NA	NA
	Decaffeinated coffee	0.71 [0.41–1.01]; (3)	NA	NA

Abbreviations: CC (colon cancer); CRC (colorectal cancer); EU (European countries); NA (not available); RC (rectal cancer).

## Supplementary Materials

The following are available online at https://www.mdpi.com/2072-6643/11/3/694/s1, PRISMA 2009 Checklist.
